# It takes a village: perceptions of Winnipeg parents, students, teachers and school staff regarding the impact of food allergy on school-age students and their families

**DOI:** 10.1186/s13223-022-00682-2

**Published:** 2022-06-10

**Authors:** Nancy Ross, Sandra Dalke, Shauna Filuk, Bev Kulbaba, Diane Marks, Jo-Anne St-Vincent, Elinor Simons

**Affiliations:** 1 Children’s Allergy & Asthma Education Centre (CAAEC), Health Sciences Centre, FE-125–685 William Avenue, Winnipeg, MB R3E 0Z2 Canada; 2 Unified Referral and Intake Service, Manitoba Health, 300 Carlton Street, Winnipeg, MB R3B 3M9 Canada; 3 Section of Allergy and Clinical Immunology, Department of Pediatrics and Child Health, University of Manitoba and Children’s Hospital Research Institute of Manitoba, 504B–715 McDermot Avenue, Winnipeg, MB R3E 3P4 Canada

## Abstract

**Background:**

The entire school community contributes to the safety of students with food allergy. We sought to determine the food allergy perceptions and education needs of parents, students and school staff, with the goal of enhancing food allergy education in schools.

**Methods:**

With ethics approval from the University of Manitoba and participating school divisions, elementary school principals emailed SurveyMonkey^®^ Questionnaire Links to their parent/caregiver contact list and school staff. We compared anonymous responses of parents of children with and without food allergy, students with and without food allergy, and parents and school staff using chi-squared tests.

**Results:**

Participants included 561 parents of school-age children (ages 7–12 years, 19% with food allergy), 61 students (23% with food allergy), and 203 school staff (62% teachers, 88% with experience managing food allergies in the classroom). Parents of children with and without food allergy considered food allergy when sending food to school (98% vs. 96%, p = 0.39). More parents of children with food allergy thought that greater information and awareness about food allergy was needed (74% vs. 44%, p < 0.0001). Students with food allergy were most interested (100%) in having other students learn not to bully and how to help during a reaction. Students without food allergy were most interested in learning how to prevent a reaction (70%). Fewer parents than school staff thought that food allergies in the classroom impacted teachers’ time (2.1% vs. 21%, p < 0.0001) and that teachers knew how to treat allergic reactions to foods (34% vs. 94%, p < 0.0001). More parents than school staff thought that banning foods from classrooms kept allergic students safe (65% vs. 34%, p = 0.006) and that having a Food Allergy Educator speak at school would be helpful (99% vs. 67%, p < 0.0001).

**Conclusions:**

Food allergy education is necessary for the entire school community and should include parents of school-aged children with and without food allergy, students with and without food allergy, and teachers and school staff. These members of the school community recognized their own and others’ needs for increased food allergy education and awareness in the school setting.

**Supplementary Information:**

The online version contains supplementary material available at 10.1186/s13223-022-00682-2.

## Background

It takes a village to raise a child and it takes the cooperation of an entire school community to keep children with food allergy safe at school. Food allergy is estimated to affect at least 6% of children [1, 2]. School-age children with food allergy and their teachers require the support and cooperation of parents and classmates with and without food allergy to prevent life-threatening reactions. Awareness, education, support, an epinephrine auto-injector, and an anaphylaxis action plan are essential to effectively manage food allergies at school [3–8]. In Manitoba, food allergy management in schools is supported by the Unified Referral and Intake Service (URIS) (https://www.gov.mb.ca/fs/childcare/resources/pubs/uris.pdf), a team of nurses who provide health care plans and training to school staff for many healthcare needs, including food allergies, at the beginning of the school year.

Few studies have evaluated the perspectives of parents of children without food allergy, students with and without food allergy, teachers and school staff. This article examines the food allergy perceptions of parents of children with and without food allergy, students with and without food allergy, and teachers and school staff regarding the impact of food allergy on the classroom and education needs of the school community.

We hypothesized that parents of children with and without food allergy, students with and without food allergy, and parents and teachers would have different perceptions about the impact of food allergy on children at school, management of food allergies at school, including banning of food allergens, and priorities for school-based anaphylaxis education. The results from this assessment will guide the development of food allergy education and awareness in schools.

## Methods

This needs assessment was developed based on discussions with parents, students, school nurses, anaphylaxis educators, and pediatric allergists. We did not identify a validated instrument that assessed the educational needs of the school community. We developed questionnaires for parents of children with and without food allergy, students with and without food allergy, and teachers and school staff to examine their experiences with food allergy, opinions about what keeps students with food allergy safe at school, and perceived needs for education.

The questionnaires focused on food allergy experiences of school-age children and their families and teachers, including:

1. Perceived positive and negative impacts of food allergy in the classroom
2. Food allergy management strategies, including food banning, and barriers to management
3. Need for food allergy education in schools, including:
  - WHO will benefit from education?
  - WHAT needs to be taught?

With ethics approval and a waiver of consent from the University of Manitoba Health Research Ethics Board, Health Sciences Centre Impact approval, and permission from the URIS, the division superintendents of school divisions in Winnipeg, Manitoba were invited to participate. Approval was obtained from interested school divisions according to their individual policies. In participating school divisions, the superintendents emailed their elementary school principals the Project Information and the SurveyMonkey^®^ Questionnaire Links for parent/caregiver (see Additional file 1), student (see Additional file 2), and teacher/school staff (see Additional file 3) questionnaires. Interested principals then emailed the SurveyMonkey^®^ Questionnaire Links to their parent/caregiver contact list and school staff. Parents were provided with links to the parent and student questionnaires and could provide the student questionnaire link to their child if they wished. All parents, school-age students, teachers, and school staff at Winnipeg elementary schools were eligible to participate if they could read and understand the questionnaires in English.

Consent was implied if individuals completed and submitted the anonymous questionnaires. Questionnaires contained no personal identifiers and identified only the school division rather than the school. There were no restrictions to participants using the same computer, so a school computer could be used by parents, students, teachers, and school staff attending information evenings. The anonymous data were collected and stored online in our SurveyMonkey^®^ account.

In order to identify priorities for teaching each group, responses were compared between parents of children with and without food allergy, between students with and without food allergy, and between parents and school staff using Chi-squared tests.

## Results

Participants included 561 parents of school-age children ages 7–12 years, 85% of whom reported banned foods at their child’s school. Foods banned in their children’s classrooms included peanut (97%), tree nuts (69%), fish (44%), shellfish (35%), egg (22%), milk (4.4%), sesame (4.4%), wheat (3.0%), and soy (2.5%). Of the 107 parents (19%) of children with food allergy, 11% reported that their child had had an allergic reaction to food at school and 5.1% were treated with an epinephrine auto-injector. Student participants included 14 (23%) with and 47 (77%) without food allergy. Participants also included 203 school staff of whom 62% were teachers. Other school staff self-identified as educational assistants (20%), office staff (5.4%), lunch supervisors (1.5%) and others (11%) including principals, early childhood educators, clinical social workers and psychologists; 88% of school staff reported experience managing food allergies in the classroom.

Most parents of school-age children with and without food allergy considered food allergy when sending food to school (98% versus 96%, p = 0.39) (Table 1). Among parents of children without food allergy, 97% considered food allergy when sending food to school among parents whose children attended a school with food bans, and 73% considered food allergy when sending food to school among parents whose children’s school did not have food bans (p < 0.0001). The comments and detailed replies indicated a variety of reasons for considering food allergy, including wanting to keep all children safe, not wanting their child to witness a severe allergic reaction, and frustration that they have to be careful about food, particularly if their child has no known contact with the child who is allergic to the food.

**Table 1:** Comparison of parents of children *with* food allergy versus *without* food allergy

Survey participants: parents of school age children (ages 7–12 years)	Parents of children *wit*h food allergy N = 107 (%^a^)	Parents of children *without* food allergy N = 454 (%^a^)	P value
Banning foods from class keeps children with food allergy safe	44 (64%)	185 (65%)	0.83
Consider food allergy when sending food to school	85 (98%)	379 (96%)	0.39
Having a child with food allergy in class teaches responsibility	23 (22%)	100 (22%)	0.90
Helps children to be aware of other’s needs	29 (54%)	25 (10%)	< 0.0001
Child with food allergy restricts other children	26 (24%)	216 (48%)	< 0.0001
Food allergy impacts teachers’ time	3 (2.8%)	9 (2.0%)	0.60
Greater awareness and information about food allergy is needed in my child’s school	48 (74%)	128 (44%)	< 0.0001
Food Allergy Educator speaking to students and staff would be helpful	61 (91%)	186 (87%)	** < **0.0001

^a^Percent excluding non-responders

Parents of children with food allergies were less likely to report classroom restrictions because of food allergies (24% versus 48%, p < 0.0001), and more likely to report that food allergies helped children to be aware of other’s needs (54% versus 10%, p < 0.0001) and that greater awareness and information about food allergies were needed at school (74% versus 44%, p < 0.0001) (Table 1).

Similar proportions of parents of school-age children with and without food allergy believed that banning foods from class kept allergic students safe (64% versus 65%, p = 0.83). (Table 1) Parents of children with and without food allergy reported similar reasons (Table 2) for supporting banning foods (p = 0.40), concern regarding the number of foods banned (p = 0.36), need to modify banning requirements depending on the children’s ages (p = 0.15), and concern that banning foods did not guarantee safety (p = 0.20). More parents of children with food allergy expressed concerns regarding poor adherence to food bans (p < 0.0001), and more parents of children without food allergy opposed banning foods (p = 0.03) (Table 2).

**Table 2:** Comments from parents of school age children: does banning allergenic foods make allergic students safe?

Responses, reasons and concerns	Parent of children *with* food allergy N = 38 (%^a^)	Parent of child *withou*t food allergy N = 141 (%^a^)	P value
Support banning foods	7 (18%)	24 (17%)	0.40
Teacher workload decreased			
Lower risk of reactions			
Request for non-food reward			
Parents take responsibility for health			
Concern with number of foods banned	5 (13%)	21 (15%)	0.36
Only some allergenic foods banned			
Picky eaters or cultural preferences			
Higher cost of allowed foods			
Restrictions in classes with no allergy			
Punishment for accidental allergens			
Issues different at different ages	8 (21%)	20 (14%)	0.15
Needs change with age			
May give a false/temporary sense of security			
Adherence concerns	19 (50%)	26 (18%)	< 0.0001
Families will not adhere to banning			
Families of children without food allergy do not avoid food allergens effectively			
Families of children without food allergy request more information			
Banning is no guarantee of safety	8 (21%)	39 (28%)	0.20
Prefer allergen aware vs. allergen free			
Previous outside consumption			
Against banning	5 (13%)	40 (28%)	0.03
Restaurant/grocery higher risk			
Ineffective/more reactions			
Prefer education/adequate cleaning			
Safety/responsibility start at home			
Support segregation by table or class			

^a^Percent excluding non-responders

Students with food allergy were most interested (100%) in having other students learn the seriousness of food allergy, not to bully, and how to help during a reaction. Two thirds of students without food allergy were interested in learning all of the topics, including preventing a reaction and the seriousness of food allergy (Table 3).

**Table 3:** Comparison of children *with* food allergy versus *without* food allergy

Survey participants: children (ages 7–12 years)	Children *with* food allergy N = 14 (%^a^)	Children *without* food allergy N = 47 (%^a^)	P value
I want to know more about food allergy	6 (46%)	14 (30%)	0.13
I want to know about			
Preventing a reaction	6 (60%)	16 (70%)	0.29
What a reaction looks like	8 (80%)	15 (65%)	0.17
Using the EpiPen	4 (40%)	14 (61%)	0.13
Telling others about food allergy	5 (50%)	6 (29%)	0.13
I want other kids in my class to know more about food allergy	12 (92%)	21 (49%)	0.002
I would like to meet kids with food allergy at my school	4 (40%)	16 (42%)	0.44
I want other kids in my class to know			
Food allergy is serious	10 (100%)	23 (72%)	0.03
Not to tease or bully someone with food allergy	10 (100%)	21 (66%)	0.01
Not to share food with someone with food allergy	9 (90%)	22 (69%)	0.09
How to help during a reaction	10 (100%)	20 (63%)	0.009
I would like a food allergy nurse to talk to my class	8 (80%)	22 (58%)	0.09
I would like a food allergy nurse to talk to my school	7 (70%)	24 (63%)	0.33

^a^Percent excluding non-responders

More parents than school staff thought that banning foods from classrooms kept allergic students safe (65% versus 34%, p = 0.006) (Table 4). Fewer parents were concerned that banning was not sufficient to keep children with food allergy safe (p = 0.002); parents were more likely to worry about the number of foods being banned (p = 0.002) (Table 5). No school staff commented on their time as a factor related to banning; all staff comments discussed the safety of their students. Fewer parents than school staff thought that food allergies in the classroom impacted teachers’ time (2.1% versus 21%, p < 0.0001) and that teachers knew how to treat allergic reactions to foods (34% versus 94%, p < 0.0001).

**Table 4:** Comparison of parents (*wit*h and *without *food allergy) versus school staff

Survey participants: teachers and school staff	Parents of children *with* & *without* food allergy N = 561 (%^a^)	School staff^a^ (61.6% teachers) N = 203 (%^a^)	P value
Banning foods from class keeps children with food allergy safe	229 (65%)	63 (34%)	0.006
Teachers know how to treat allergic reactions	169 (34%)	174 (94%)	< 0.0001
Food allergy impacts teachers’ time	12 (2.1%)	22 (21%)	< 0.0001
Greater food allergy awareness and information is needed in my school	176 (50%)	54 (30%)	0.06
Food Allergy Educator speaking to students and staff would be helpful	247 (99%)	80 (67%)	< 0.0001
Consider food allergy when sending food to school	464 (94%)	166 (91%)	0.07
Helps children to be aware of other’s needs	102 (35%)	86 (82%)	< 0.0001
Child with food allergy restricts activities	242 (82%)	84 (80%)	0.29

^a^Percent excluding non-responders

**Table 5:** Comments from parents and teachers of school age children: does banning allergenic foods make allergic students safe?

Responses, reasons and concerns	Parents of children *with and without* food allergy N = 179 (%^a^)	Teachers and school staff N = 90 (%^a^)	P value
Support banning foods	31 (17%)	13 (14%)	0.26
Teacher workload decreased			
Lower risk of reactions			
Request for non-food reward			
Parents take responsibility for health			
Concern with number of foods banned	26 (14%)	3 (3%)	0.002
Only some allergenic foods banned			
Picky eaters or cultural preferences			
Higher cost of allowed foods			
Restrictions in classes with no allergy			
Punishment for accidental allergens			
Issues different at different ages	28 (16%)	12 (13%)	0.29
Needs change with age			
May give a false/temporary sense of security			
Adherence concerns	45 (25%)	26 (29%)	0.23
Families will not adhere to banning			
Families of children without food allergy do not avoid food allergens effectively			
Families of children without food allergy request more information			
Banning is no guarantee of safety	47 (26%)	39 (43%)	0.002
Prefer allergen aware vs. allergen free			
Previous outside consumption			
Against banning	45 (25%)	16 (18%)	0.09
Restaurant/grocery higher risk			
Ineffective/more reactions			
Prefer education/adequate cleaning			
Safety/responsibility start at home			
Support segregation by table or class			

^a^Percent excluding non-responders

**Fig. 1:**
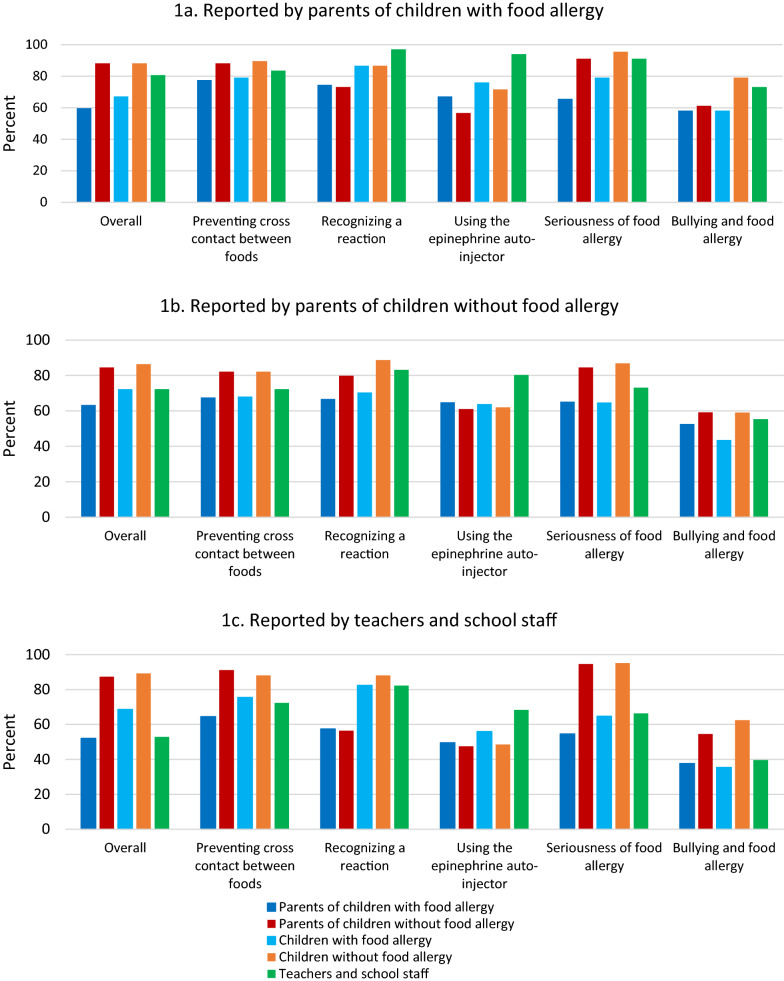
Who should receive education and what topics should be covered? **a** Reported by parents of children with food allergy. **b** Reported by parents of children without food allergy. **c **Reported by teachers and school staff

Parents of children with and without food allergy and school staff all reported an overall need for more food allergy education in schools (Fig. 1). All three groups wanted more education for themselves but recognized a particular need for more education of parents of children without food allergy and students without food allergy. Parents of children without food allergy recognized their own need for more education regarding preventing cross contact between foods, recognizing a reaction, seriousness of food allergy, and bullying around food allergy. Teachers rated themselves and their students with and without food allergy as needing education regarding recognizing a reaction and rated themselves as needing education regarding administering the epinephrine auto-injector.

## Discussion

The need for a coordinated approach to food allergy management in schools has long been recognized to improve the physical and emotional safety for children with food allergy at school [3, 9–12]. Of the medical conditions for which students require accommodation at school, food allergy is one of the few that requires parents of children without the condition to modify their activities at home to keep their child’s classmates safe [4, 11, 13].

Our findings support the importance of engaging parents of children without food allergy and students without food allergy in education regarding food allergies. Parents are often required to support an Allergen Aware or Nut Free Environment at their child’s school with the explanation that there are children with life-threatening food allergies in the classroom. In many instances, these parents have received a letter from the school requesting avoidance of certain food allergens but have not received additional training regarding food allergies or label reading. Parental attitudes towards foods allergies may also influence their child’s perceptions. Published studies highlight misconceptions regarding food allergy for people without food allergy experience, including potential triggers and severity [4, 13, 14].

Although banning highly allergenic foods from elementary schools is no longer recommended, most parents reported banning of allergenic foods at their child’s school. Parents and teachers had varied opinions about and concerns with banning. More parents of children with food allergy reported a need for improved knowledge and motivation of parents of children without food allergy to correctly avoid providing banned foods and expressed the differing needs at different levels of child development. Similar proportions of parents of children with and without food allergy expressed concern regarding the increasing numbers of foods banned. Parents of children with food allergy also described situations where banning foods created conflicting needs, such as nut bans preventing a child with cow’s milk allergy from having a nut-based milk at school, while cow’s milk might or might not be banned from the classroom.

Both parents and teachers expressed concern that banning was not enough to keep children with food allergy safe, although an additional 14% of parents of children without food allergy considered food allergy when sending food to school if their child attended a school with food bans. Teachers were more concerned that banning was not sufficient to keep children with food allergy safe; parents were more likely to worry about the number of foods being banned.

A large Canadian study of 1941 children with peanut allergy reported a 12.4% annual incidence of accidental exposure to peanut and no difference between daycares and schools that banned peanut versus those that permitted peanut [5]. Although issues around banning foods still cause emotional responses from parents, published recommendations no longer recommend banning of food allergens, except in particular circumstances [3]. Given the absence of data on school food bans increasing safety of students with food allergy and the degree of concern regarding food bans, education regarding allergen avoidance and the need for banning foods, depending on the context, may be of benefit to school communities.

Participants reported bullying of children with food allergy because of their food allergy and bullying of children without food allergy who inadvertently brought a banned food to school. Bullying was recognized by parents of children with and without food allergy as a topic needing to be addressed by food allergy education. These education requests are supported by the literature, which shows that children with food allergy frequently report anxiety and bullying [15–17] and are more likely to face bullying than those without food allergy [18].

In this study, a substantial portion of parents of children without food allergy and students without food allergy reported that they needed education, suggesting that they were interested in and recognized their need for education regarding food allergy in schools. Parents of school-age children with and without food allergy agreed regarding aspects of food allergy management that contributed to the safety of children with food allergy. Most parents reported that children with and without food allergy needed education regarding food allergy, although a higher proportion in both groups thought that children without food allergy needed education. Parents of students with and without food allergy also wanted additional Food Allergy Educator support for school staff.

Teachers and school staff reported greater confidence in their ability to treat reactions at school than parents believed, although they also reported their own need for more education. In an electronic survey of 724 Canadian teachers, 80% reported that they were confident in recognizing, responding to, and treating anaphylaxis with an epinephrine auto-injector [19]. In a Quebec study, 343 teachers and school staff with anaphylaxis training reported confidence in using an epinephrine auto-injector but performed poorly when demonstrating use of an EpiPen^®^ [20]. Our results extend the findings of these studies and showed that teachers and school staff recognized their need for further education.

Food allergy education is necessary for the entire school community and should include parents of school-aged children with and without food allergy and school staff [3, 4]. If parents of children without food allergy are provided with more context about severity and triggers this may translate to normalization of food allergy and possibly less bullying [9]. Parents influence the belief system of their children and food allergy education for parents of children without food allergy will contribute to the education of children without food allergy. Children and parents of children without food allergy need to be included in further research regarding education needs in schools.

Strengths of this study include the questionnaire responses of over 500 parents and 200 teachers and school staff, and inclusion of large numbers of parents of children without food allergy. Generalizability of the study may be limited by the relatively high proportion of parents of children with food allergy and students with food allergy compared to the general population, and the high proportion of schools using allergenic food banning as an allergen avoidance strategy. Limitations also include the exclusion of families unable to respond in English, the relatively small number of students participating, the lack of information about parents, students and teachers who did not respond, and the anonymous nature of the questionnaires, which precluded linking related parent, student and teacher groups and recalling participants to ask further questions.

## Conclusions

Food allergy education is necessary for the entire school community and should include parents of school-aged children with and without food allergy, students with and without food allergy, and teachers and school staff. In our study, these members of the school community recognized their own and others’ need for increased food allergy education and awareness in the school setting, in order to improve the safety and support of students with food allergy. This assessment has identified areas of need for food allergy education and awareness in the school setting and will guide the future development of food allergy education and awareness in schools.

## Supplementary Information

**Additional file 1.** Parent/Caregiver Questionnaire.

**Additional file 2.** Student Questionnaire.

**Additional file 3.** Teacher/School Staff Questionnaire.

## Data Availability

The datasets analysed during this study are available from the corresponding author on reasonable request.

## Abbreviation

URIS: Unified Referral and Intake Service
