# Supplementary material for: It takes a village: perceptions of Winnipeg parents, students, teachers and school staff regarding the impact of food allergy on school-age students and their families
Source: Allergy Asthma Clin Immunol. 2022 Jun 10;18:47. doi: 10.1186/s13223-022-00682-2 (PMC9188203; doi:10.1186/s13223-022-00682-2)
Supplement: Supplementary file 1 — Additional file 1. Parent/Caregiver Questionnaire. [file 13223_2022_682_MOESM1_ESM.pdf]

children's allergy & asthma education centre

This survey is being conducted by the staff at the Children's Allergy & Asthma Education Centre (www.caaec.ca) at the Children's Hospital in Winnipeg. This survey is intended for parents in the Winnipeg area who have children at an elementary school. We would like your feedback on food allergies at school. The information you provide will help us to better understand the needs of students with and without food allergies at school. We will use this information to develop a food allergy education and awareness program for children with food allergies and their classmates and teachers.

We will not collect any information in the survey that tells us who you are. However, once you complete the survey, we will provide the CAAEC email address and you may send us your email address for a chance to win 1 of 2 Indigo Chapter Gift Cards worth $50 each.

If you have any questions about the survey, please contact Nancy Ross RN BN at caaec@hsc.mb.ca .

If you agree to take part in the survey please begin.

* 1. This survey is for parents of children with or without food allergy.
Do you have a child with food allergy?

◯ Yes

◯ No

children's allergy & asthma education centre

* 2. How many children with a life threatening food allergy diagnosed by an Allergist do you have in your family?

- ◯ 0
- ◯ 1
- ◯ 2
- ◯ 3
- ◯ 4 or more children

children's allergy & asthma education centre

3. What are the ages of children in your family with food allergy? (mark all that apply)

- [ ] 0-4 years old
- [ ] 5-6 years old
- [ ] 7-12 years old
- [ ] 13 years and older

4. We have a few more questions about your child/children with food allergies.
How was/were foodallergy/allergies identified?
(mark all that apply)

- [ ] Reactions or symptoms to the food
- [ ] Positive skin prick test
- [ ] Positive blood test
- [ ] Other (please specify)

5. To what food(s) is your child/children allergic? (mark all that apply)

- [ ] Peanut
- [ ] Tree Nuts
- [ ] Milk
- [ ] Egg
- [ ] Shellfish
- [ ] Fish
- [ ] Soy
- [ ] Wheat
- [ ] Sesame

Other (please specify)

6. What were the signs or symptoms of your child's reaction? (mark all that apply)

- [ ] Severe itching
- [ ] Hives/rash
- [ ] Flushing or redness
- [ ] Swelling of lips
- [ ] Swelling of tongue
- [ ] Other swelling
- [ ] Hard to swallow
- [ ] Hoarseness
- [ ] Coughing
- [ ] Difficult or noisy breathing
- [ ] Eyes watering or redness
- [ ] Nose running
- [ ] Diarrhea
- [ ] Vomiting
- [ ] Dizziness or feeling faint
- [ ] Passing out
- [ ] Other (please specify)

7. Does/do your child/children have a written anaphylaxis plan at school?

- ( ) Yes
- ( ) No
- ( ) Not Sure

8. Does the written anaphylaxis plan outline how to reduce risks of a reaction?

- ( ) Yes
- ( ) No
- ( ) Not sure

9. Can your child/children recognize a reaction?

- ◯ Yes
- ◯ No
- ◯ Not sure

10. Does your child refuse food if they are not sure if the food is safe?
(e.g. Baked treats are brought to school for a class party with no ingredient list).

- ◯ My child/children would always refuse food
- ◯ My child/children would sometimes refuse food
- ◯ My child/children would never refuse food
- ◯ Not sure

11. Could your child/children use their EpiPen if they had to?

- ◯ Yes, with help
- ◯ Yes, without help
- ◯ No
- ◯ Not sure

12. Has/have your child/children had a reaction at school?

- ◯ Yes
- ◯ No
- ◯ Not sure

13. Has/have your child/children ever been given an EpiPen at school to treat a reaction?

- ☐ Yes
- ☐ No
- ☐ Not sure

Other (please specify)

[ ]

14. Does/do your child/children feel safe with their food allergy at school?

- All of time
- Most of the time
- Some of the time
- None of the time
- Not sure

15. Are there students/other students with food allergy in your child's class?

- Yes
- No
- Not sure

16. Are certain foods banned from your child's class?

- Yes
- No
- Not sure

children's allergy & asthma education centre

17. Please list the foods that are not allowed in the class? (mark all that apply)

- [ ] Peanut
- [ ] Tree Nuts
- [ ] Milk
- [ ] Egg
- [ ] Shellfish
- [ ] Fish
- [ ] Soy
- [ ] Wheat
- [ ] Sesame

Other (please specify)

____

18. Does banning foods keep food allergic students safe?

- Yes
- No
- Not sure
- Comments

19. Does your child's teacher know how to recognize an allergic reaction?

- Yes
- No
- Not sure

20. Does your child's teacher know how to respond to/treat an allergic reaction?

- Yes
- No
- Not sure

21. Do other students in the class know how to keep a student with food allergies safe from a reaction?

- Yes
- No
- Not sure

22. Does having a child/other child with food allergies in the class impact your child?

- Yes
- No
- Not sure

children's allergy & asthma education centre

23. How does having a child/ other child with food allergies in the class impact your child?

- ☐ Helps my child know they are not the only one with food allergies
- ☐ Helps my child know they are not the only one living with a health problem
- ☐ Restricts what food my child can bring to school
- ☐ Teaches responsibility
- ☐ Takes up the teacher's time
- ☐ Other (please specify)

____

24. Do you consider other students' food allergies when sending food to school?

- ○ Yes
- ○ No
- ○ Not sure

25. Is more information and awareness about food allergies needed in your child's school?

- ○ Yes
- ○ No
- ○ Not sure

children's allergy & asthma education centre

26. Who could benefit from more information and awareness? (mark all that apply)

- [ ] Students with allergies
- [ ] Students without allergies
- [ ] Teachers
- [ ] Educational assistants
- [ ] Office staff
- [ ] Lunch supervisors
- [ ] Parents of students with allergies
- [ ] Parents of students without allergies
- [ ] Not sure
- [ ] Other (please specify)

27. For students *with allergies* what topics would be helpful? (mark all that apply)

- [ ] Preventing cross contact between foods
- [ ] Recognizing a reaction
- [ ] Using the EpiPen
- [ ] Seriousness of food allergy
- [ ] Bullying and food allergy
- [ ] Not sure
- [ ] Other (please specify)

28. For students *without allergies* what topics would be helpful? (mark all that apply)

- [ ] Preventing cross contact between foods
- [ ] Recognizing a reaction
- [ ] Using the EpiPen
- [ ] Seriousness of food allergy
- [ ] Bullying and food allergy
- [ ] Not sure
- [ ] Other (please specify)

29. For *teachers/school staff* what topics would be helpful? (mark all that apply)

- [ ] Preventing cross contact of foods
- [ ] Recognizing a reaction
- [ ] Using the EpiPen
- [ ] Seriousness of food allergy
- [ ] Bullying and food allergy
- [ ] Not sure
- [ ] Other (please specify)

30. For *parents of children with allergies* what topics would be helpful? (mark all that apply)

- ☐ Preventing cross contact between foods
- ☐ Recognizing a reaction
- ☐ Using the EpiPen
- ☐ Seriousness of food allergy
- ☐ Bullying and food allergy
- ☐ Not sure
- ☐ Other (please specify)

31. For *parents of children without allergies* what topics would be helpful? (mark all that apply)

- ☐ Preventing cross contact between foods
- ☐ Recognizing a reaction
- ☐ Using the EpiPen
- ☐ Seriousness of food allergy
- ☐ Bullying and food allergy
- ☐ Not sure
- ☐ Other (please specify)

32. Would it be helpful to have a food allergy educator speak to staff and students at the school?

- ○ Yes
- ○ No
- ○ Not sure

33. Are there food allergy resources you would like to see in the class?

- ○ Yes
- ○ No
- ○ Not sure

Suggestions

34. My child's school division is:

35. Is there anything you would like to add on the topic of food allergies in schools?

Thank you for completing the survey. If you would like a chance to win 1 of 2 Indigo/Chapters gift card worth $50, please email us at caaec@hsc.mb.ca and in the subject line write "parent survey". We will not use your email address for any other reason and will delete your address once the draw is complete.
