# Supplementary material for: It takes a village: perceptions of Winnipeg parents, students, teachers and school staff regarding the impact of food allergy on school-age students and their families
Source: Allergy Asthma Clin Immunol. 2022 Jun 10;18:47. doi: 10.1186/s13223-022-00682-2 (PMC9188203; doi:10.1186/s13223-022-00682-2)
Supplement: Supplementary file 2 — Additional file 2. Student Questionnaire. [file 13223_2022_682_MOESM2_ESM.pdf]

Hello. This survey is being run by the Children's Allergy & Asthma Education Centre (www.caaec.ca) at the Children's Hospital in Winnipeg.

This survey is for students with food allergy ages 7 to 12 years old.

We would like your ideas about food allergy in school.

We do not need to know your name and will not know who answered these questions.

When you are done, if you want, ask your parent to send us their email address and you will have a chance to win a Toys R Us Gift Card for $50.

If you have any questions about the survey please have your parent contact Nancy Ross RN BN CAE at caaec@hsc.mb.ca

If your parent and you agree to take part in the survey please begin.

1. How old are you?

2. Do you have a food allergy?

- Yes
- No

3. My food allergy is: (mark all that apply)

- [ ] Peanut
- [ ] Treenuts
- [ ] Milk
- [ ] Egg
- [ ] Shellfish
- [ ] Fish
- [ ] Soy
- [ ] Wheat
- [ ] Sesame
- [ ] Other (please type)

4. Do you have an EpiPen?

- ( ) Yes
- ( ) No
- ( ) Not sure

5. Where is your EpiPen at school?

- [ ] I carry it on me
- [ ] In the office
- [ ] In the classroom
- [ ] With the teacher
- [ ] Not sure
- [ ] Other (please specify)

6. Does your teacher help you with your food allergy at school?

- ◯ Yes
- ◯ No
- ◯ Not sure

7. Does your teacher know what to do if you have a reaction?

- ◯ Yes
- ◯ No
- ◯ Not sure

8. Does your school have a written plan for your food allergies?

- ◯ Yes
- ◯ No
- ◯ Not sure

9. Please type in your answer.
What is the hardest thing about having a food allergy at school?

10. Do you worry about food allergy at school?

- ◯ No, I don't worry about it
- ◯ Yes, I worry some of the time
- ◯ Yes, I worry most of the time
- ◯ Yes, I worry all the time

11. When do you worry about food at school? (mark all that apply)

- [ ] In the classroom
- [ ] At lunchtime
- [ ] At recess
- [ ] On field trips
- [ ] With class parties
- [ ] Other (please type)

12. Please type in your answer.
What are some things that would help you with food allergy at school?

13. Would you like to know more about food allergies?

- ○ Yes
- ○ No
- ○ Not sure

14. I want to know more about: (mark all that apply)

- [ ] Preventing a reaction
- [ ] What a reaction looks like
- [ ] Using the EpiPen
- [ ] Telling others about food allergy
- [ ] Other (please type)

15. I want other kids in my class to know more about food allergies:

- ( ) Yes
- ( ) No
- ( ) Not sure

16. I want other kids in my class to know: (mark all that apply)

- [ ] Food allergy is serious
- [ ] Not to tease someone with food allergy
- [ ] Don't share food with me
- [ ] How to help me if I have a reaction
- [ ] Other (please type)

17. Would you like to meet other kids at your school with food allergy?

- ( ) Yes
- ( ) No
- ( ) Not sure

18. Would you like a food allergy nurse to talk to*your class*?

- ( ) Yes
- ( ) No
- ( ) Not sure

19. Would you like a food allergy nurse to talk to*everyone at the school?*

- ( ) Yes
- ( ) No
- ( ) Not sure

20. In what school division is your school?

Thank you for completing the survey.
If you would like a chance to win a **$50 Toys R Us Gift Card**, please have your parent send us an email at **caaec@hsc.mb.ca** and write "student survey" in the subject line. Winners will be contacted by email. We will not use your email for any other reason.
Thank you.
