# Supplementary material for: It takes a village: perceptions of Winnipeg parents, students, teachers and school staff regarding the impact of food allergy on school-age students and their families
Source: Allergy Asthma Clin Immunol. 2022 Jun 10;18:47. doi: 10.1186/s13223-022-00682-2 (PMC9188203; doi:10.1186/s13223-022-00682-2)
Supplement: Supplementary file 3 — Additional file 3. Teacher/School Staff Questionnaire. [file 13223_2022_682_MOESM3_ESM.pdf]

**This survey is being conducted the staff at the Children's Allergy & Asthma Education Centre (www.caaec.ca) at the Children's Hospital in Winnipeg. We would like to know what you think about how to manage food allergies at school and keep children with food allergies safe. The information you provide will help us to better understand the needs of students with and without food allergies at school. We will use this information to develop a food allergy education and awareness program for children with food allergies, and their classmates and teachers.**

**This survey is intended for Teachers and School staff who work in Winnipeg schools attended by 5-12 year old students with a food allergy.**
**We will not collect any information in the survey that tells us who you are. However, once you complete the survey, we will provide the Allergy Centre email address and you can send us your email address for a chance to win 1 of 2 Indigo Chapter Gift Cards worth $50 each.**

**If you have any questions about the survey, please contact Nancy Ross RN BN at caaec@hsc.mb.ca**

**If you agree to take part in the survey please begin.**

* 1. Information about you:
I am a

- ◯ Teacher
- ◯ Educational Assistant
- ◯ Resource Teacher
- ◯ Office staff
- ◯ Lunch room supervisor
- ◯ Other school staff member
- ◯ Other (please specify)

[ ]

2. I have experience working with students who have food allergies and have been prescribed an EpiPen

- ◯ Yes
- ◯ No

3. Are there students with food allergies at your school?

- ◯ Yes
- ◯ No
- ◯ Not sure

4. What food allergies have students in your school had? (mark all that apply)

- [ ] Peanut
- [ ] Tree Nuts
- [ ] Milk
- [ ] Egg
- [ ] Shellfish
- [ ] Fish
- [ ] Soy
- [ ] Wheat
- [ ] Sesame
- [ ] None

Other (please specify)

5. Do students with food allergy have a written anaphylaxis plan at your school?

- ○ None
- ○ Some
- ○ All
- ○ Not sure

6. Are certain foods banned from your school?

- ○ Yes
- ○ No
- ○ Not sure

7. Please list the foods that are not allowed in school. (mark all that apply)

- [ ] Peanut
- [ ] Tree Nuts
- [ ] Milk
- [ ] Egg
- [ ] Shellfish
- [ ] Fish
- [ ] Soy
- [ ] Wheat
- [ ] Sesame

Other (please specify)

8. Does banning foods keep food allergic students safe?

- ○ Yes
- ○ No
- ○ Not sure
- ○ Comments

9. Do school staff know how to keep students with food allergies safe from a reaction?

- ○ Yes
- ○ No
- ○ Not sure

10. Do school staff know how to respond to/treat a reaction?

- ○ Yes
- ○ No
- ○ Not sure

11. Do students know how to keep a student with food allergies safe from a reaction?

- ◯ Yes, all of the time
- ◯ Most of the time
- ◯ Some of the time
- ◯ No
- ◯ Not sure

12. Do *students with food allergy* know how to keep themselves safe from a reaction?

- ◯ Yes, all of the time
- ◯ Most of the time
- ◯ Some of the time
- ◯ No
- ◯ Not sure

13. Does having a student with food allergy impact your classroom?

- ◯ Yes
- ◯ No
- ◯ Not sure
- ◯ Does not apply

14. How does it impact your classroom?

- [ ] Helps students be more aware of others needs
- [ ] Takes up time
- [ ] Impacts field trip planning
- [ ] Impacts class parties
- [ ] Other (please specify)

15. Do you consider food allergies when bringing food into your classroom?

- Yes
- No
- Not sure
- Does not apply

16. Is more information and awareness about food allergies needed in your school?

- Yes
- No
- Not sure

17. Who could benefit from more information and awareness? (mark all that apply)

- [ ] Students *with* allergies
- [ ] Students *without* allergies
- [ ] Teachers
- [ ] Educational assistants
- [ ] Office staff
- [ ] Lunch supervisors
- [ ] Parents of students *with* allergies
- [ ] Parents of students *without* allergies
- [ ] Not sure
- [ ] Other (please specify)

18. For ***students with allergies,*** what topics would be helpful? (mark all that apply)

- ☐ Preventing cross contact between foods
- ☐ Recognizing a reaction
- ☐ Using the EpiPen
- ☐ Seriousness of food allergy
- ☐ Bullying and food allergy
- ☐ Telling others about their food allergy
- ☐ Carrying their EpiPen
- ☐ Not sure
- ☐ Other (please specify)

19. For *students without allergies* what topics would be helpful? (mark all that apply)

- ☐ Preventing cross contact between foods
- ☐ Recognizing a reaction
- ☐ Using the EpiPen
- ☐ Seriousness of food allergy
- ☐ Bullying and food allergy
- ☐ Not sure
- ☐ Other (please specify)

20. For *school staff* what topics would be helpful? (mark all that apply)

- [ ] Preventing cross contact between foods
- [ ] Recognizing a reaction
- [ ] Using the EpiPen
- [ ] Seriousness of food allergy
- [ ] Bullying and food allergy
- [ ] Not sure
- [ ] Other (please specify)

21. For *parents of children with food allergies* what topics would be helpful? (mark all that apply)

- [ ] Preventing cross contact between foods
- [ ] Recognizing a reaction
- [ ] Using the EpiPen
- [ ] Seriousness of food allergy
- [ ] Bullying and food allergy
- [ ] Not sure
- [ ] Other (please specify)

22. For *parents of children without allergies*, what topics would be helpful? (mark all that apply)

- [ ] Preventing cross contact between foods
- [ ] Recognizing a reaction
- [ ] Using the EpiPen
- [ ] Seriousness of food allergy
- [ ] Bullying and food allergy
- [ ] Not sure
- [ ] Other (please specify)

23. Would it be helpful to have a food allergy educator speak to staff and students at your school?

- Yes
- No
- Not sure

24. Are there food allergy resources you would like to see in your class or school?

- Yes
- No
- Not sure

Suggestions

25. My school division is:

26. Is there anything you would like to add on the topic of food allergies in schools?

Thank you for completing the survey. If you would like a chance to win 1 of 2 Indigo/Chapters gift card worth $50, please email us at caaec@hsc.mb.ca and in the subject line write "Teacher/School Staff". We will not use your email address for any other reason and will delete your address once the draw is complete.
